# PLA-index: A *k*-mer Index Exploiting Rank Curve Linearity

**DOI:** 10.4230/LIPIcs.WABI.2024.13

**Published:** 2024-08-26

**Authors:** Hasin Abrar, Paul Medvedev

**Affiliations:** Department of Computer Science and Engineering, The Pennsylvania State University, University Park, PA, USA; Department of Computer Science and Engineering, The Pennsylvania State University, University Park, PA, USA; Department of Biochemistry and Molecular Biology, The Pennsylvania State University, University Park, PA, USA; Huck Institutes of the Life Sciences, The Pennsylvania State University, University Park, PA, USA

**Keywords:** K-mer index, Piece-wise linear approximation, Learned index, Applied computing → Bioinformatics, Applied computing → Computational biology, Theory of computation → Data structures design and analysis

## Abstract

Given a sorted list of *k*-mers *S*, the rank curve of *S* is the function mapping a *k*-mer from the *k*-mer universe to the location in *S* where it either first appears or would be inserted. An exciting recent development is the observation that, for certain datasets, the rank curve is predictable and can be exploited to create small search indices. In this paper, we develop a novel search index that first estimates a *k*-mer’s rank using a piece-wise linear approximation of the rank curve and then does a local search to determine the precise location of the *k*-mer in the list. We combine ideas from previous approaches and supplement them with an innovative data representation strategy that substantially reduces space usage. Our PLA-index uses an order of magnitude less space than Sapling and uses less than half the space of the PGM-index, for roughly the same query time. For example, using only 9 MiB of memory, it can narrow down the position of *k*-mer in the suffix array of the human genome to within 255 positions. Furthermore, we demonstrate the potential of our approach to impact a variety of downstream applications. First, the PLA-index halves the time of binary search on the suffix array of the human genome. Second, the PLA-index reduces the space of a direct-access lookup table by 76 percent, without increasing the run time. Third, we plug the PLA-index into a state-of-the-art read aligner Strobealign and replace a 2 GiB component with a PLA-index of size 1.5 MiB, without significantly effecting runtime. The software and reproducibility information is freely available at https://github.com/medvedevgroup/pla-index.

## 1 Introduction

Modern biological sequence analysis is often performed at the level of *k*-mers – strings of a fixed length *k*. Datasets are stored as collections of *k*-mers, whether they come from a set of reads or an assembled genome or transcriptome. For methods working with such data, it becomes essential to efficiently determine if a *k*-mer belongs to a collection or not. The design of an index to store and query a collection of *k*-mers has thus been a central area of research and progress in algorithmic bioinformatics in the last decade [5, 17]. In this paper, we are particularly interested in what we call a *search index*, which assumes that the collection is stored sorted in memory and a search index supplements it in order to allow fast location queries.

**Figure 1:**
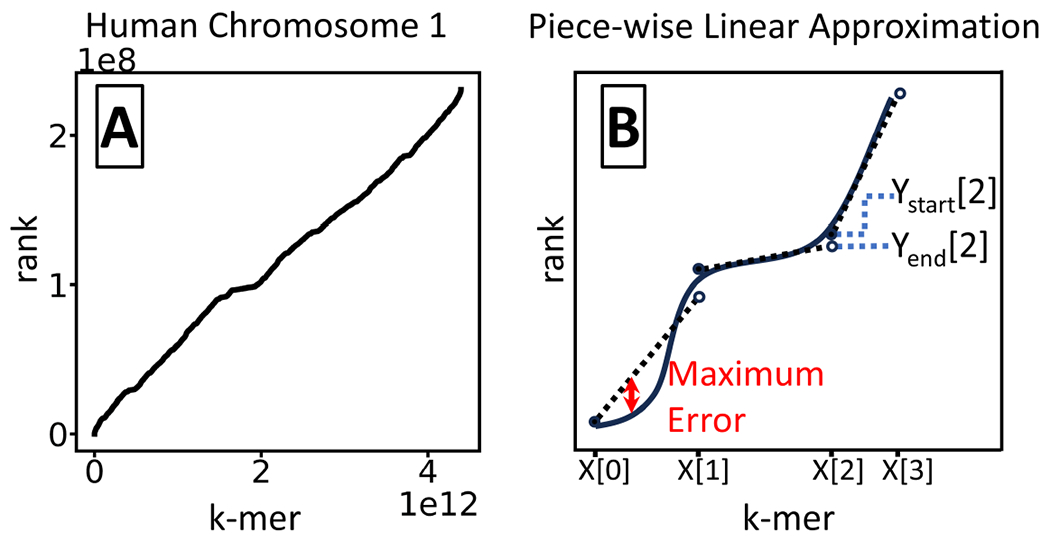
Panel A shows the rank curve for the sorted list of all constituent 21-mers of human chr1. The curve can deviate from the straight line for two general reasons: vertical jumps due to repetitive *k*-mers and horizontally flat parts due to long lexicographical stretches of *k*-mers that do not appear in the genome. The intuition is that as long as these effects happen roughly uniformly along the curve, the curve can be approximated with only a few segments. Panel B shows a cartoon illustration of a rank curve (solid black curve) and a piece-wise linear approximation (dashed black lines).

An exciting recent innovation in the broader Computer Science community has been to exploit the regularity of dataset’s rank curve in order to construct learned search indices [16]. Given a sorted list of elements from an ordered universe, the rank function takes an element *x* and returns the smallest location *i* such that all elements before *i* are smaller than *x* (see Figure 1A for an example). If *x* is in the list, this is simply the first location of *x* in the list. It was hypothesized in [16] that the rank curves of real world datasets are often highly predictable. Follow up work [10] showed that in fact one can approximate the rank curve using a piece-wise linear approximation (PLA) with a surprisingly few number of segments [10, 9, 15] (see Figure 1B for an example). PLA-based approaches have been applied to construct dictionaries [3], monotone minimal perfect hash functions [8], and search indices [10, 9, 15]. These recent innovations hold much promise for genomic datasets, where they were used to speed up suffix array search [15] and sequence alignment [12, 13, 14].

For search indices, there is a natural construction based on storing a piece-wise linear approximation to the rank curve. For a query *x*, one can compute the value of the PLA at *x* and then perform a local search in the list around the predicted location to determine if *x* is present or not. The state-of-the-art search index that follows this paradigm is the PGM-index [9], though it was not applied to the *k*-mer list setting. On the other hand, the only work that applies the PLA paradigm to the *k*-mer list setting (Sapling [15]) does not exploit its full power. In particular, Sapling limits the PLA to have fixed segment lengths, constrains adjacent segments to be continuous, and does not enforce a maximum prediction error. Nevertheless, they demonstrate how a PLA-based search index can be used to significantly accelerate suffix array lookups. Further applications remain to be found.

In this paper, we present the *PLA-index*, a search index for *k*-mer lists. Our index contains two major innovations with respect to the PGM-index [9] and Sapling [15]. The first is a novel compact representation of the PLA, achieved by modifying the construction algorithm of [9] and proving properties of the resulting segments.^1^ The second is a novel technique called repeat stretching, which reduces the number of segments in the PLA by exploiting the presence of repeats in a genome.

We show that the PLA-index is very small, e.g. using only 2 MiB of memory (respectively, 34 MiB), it can narrow down the position of *k*-mer in the suffix array of the human genome to within ±1023 positions (respectively, ±63 positions). For 34 MiB, this roughly halves the time of binary search on the suffix array. On the *k*-spectrum of the human genome, the PLA-index uses an order of magnitude less space than Sapling and uses less than half the space of the PGM-index (when holding the query time constant). We show that these results hold for various values of *k* and for other non-human genomes.

We further demonstrate how the PLA-index can be applied to improve several other downstream methods. First, we consider applications where the query time is a higher priority than lower memory, which is currently best achieved with a direct access table. The PLA-index reduces the total space by 76 %, without increasing the run time. Second, we consider a state-of-the-art short read aligner (Strobealign [26]) and show how a 2 GiB component of the aligner index can be replaced with a PLA-index of size 1.5 MiB, without significantly effecting runtime. These results demonstrate the wide applicability and potential impact of the PLA-index on *k*-mer-based methods.

## 2 Preliminaries

### *K*-mer lists

Let S denote a list of *k*-mers, sorted in non-decreasing order and indexed from 0. We let N denote the length of S and we let n denote the number of distinct *k*-mers in S. Given a string, its *sorted k-spectrum* is the sorted list of its constituent *k*-mers. For example, the 2-spectrum of the string GCCACC is S=(AC,CA,CC,CC,GC), with N=5 and n=4. For a *k*-mer x∈S, we define rank(S,x) as the largest integer 0≤i<N such that, for all 0≤j<i,S[j] is strictly less than *x*. For x∉S, we define rank(S,x)=−1. In our example, rank(S,CC)=2,rank(S,AC)=0,andrank(S,AG)=−1.

### Operations

Given a sorted list of *k*-mers S and a *k*-mer *x*, our indices will support two operations:

- search(*x*) returns a value *i* such that S[i]=x if x∈S and i=−1 otherwise, and
- rank(*x*) returns rank(S,x). ^2^

The two operations are very similar and any answer to rank is also a valid answer to search. We separate the two because there will be cases (described in Section 4) when the additional overhead of finding the first occurrence of *x* makes rank more expensive to support than search.

Indices to support these operations involve an inherent space/time trade-off. Our PLA-index and its variations will fall in between the following two extremes. On one extreme, binary search does not need any space for an index but is considered slow because it does not have cache locality. At the other extreme, one can construct a minimal perfect hash function (MPHF), with the set of keys being the distinct *k*-mers in S. An MPHF is a data structure that maps elements of the key set of size n to an integer between 0 and n−1, without any collisions. For *k*-mers that are not in the key set, the MPHF maps them to an arbitrary integer between 0 and n−1. With an MPHF, we can construct a direct-access table (i.e. an array) of size n to store the exact rank of each *k*-mer. Such a solution gives very fast queries but uses Θ(nlgN) space.

We stress that our indices are not dictionaries, i.e. they are data structures stored on top of S and do not replace S itself. Our work is thus orthogonal to the PLA-based dictionary of [3]. Similarly, our work is orthogonal to a recent paper that uses a PLA-approximation to construct a monotone MPHF [8], where the MPHF is intended to replace S and does not handle duplicates in S.

### Piece-wise linear function

A *piece-wise linear function* is defined by an array of *k*-mers X, sorted in increasing order, and two arrays $Y_{\text{start}}$ and $Y_{\text{end}}$ of y-values. Intuitively, X[i] is the x-value at the *i*^th^ breakpoint of the function, $Y_{\text{start}}[i]$ is the y-value of the segment starting at the *i*^th^ breakpoint, and $Y_{\text{end}}[i]$ is the y-value of the segment ending at the *i*^th^ breakpoint (Figure 1B). We let b=|X|−1, i.e. the number of segments. Formally, for 0≤i<b, the *i*^th^ line segment connects the points $\left(X[i],Y_{\text{start}}[i]\right)$ and $\left(X[i+1],Y_{\text{end}}[i+1]\right)$. It has slope $m_{i}=\frac{Y_{\text{end}}[i+1]-Y_{\text{start}}[i]}{X[i+1]-X[i]}$. To evaluate the function at a *k*-mer *x*, we first find the largest integer *i* such that X[i]≤x and then evaluate *x* using linear interpolation along the *i*^th^ segment. Formally,

(1) $$\text{plaEst}(x)=Y_{\text{start}}[i]+m_{i}(x-X[i]).$$

### O’Rourke’s algorithm

To compute the piece-wise linear approximation of a rank curve, we make use of an algorithm published by O’Rourke [19] and implemented in [3]. The input to the algorithm is presented in an on-line manner, i.e. one element at a time. Each element consists of an x-value *x* and a y-range [ℓ,h]. It is required that *x* is strictly larger than previous x-values. A line is said to fit these points if for each *x*, its y-value lies in the range [ℓ,h]. The algorithm maintains the set of all lines that fit the input so far^3^. When an input element is presented such that there is no longer any line that fits the data, the algorithm outputs a line that fits the previous ranges and terminates. The algorithm running time and memory is linear in the number of input elements.

## 3 The basic PLA-index

The basic PLA-index is constructed from S and an error threshold ε. It consists of a piece-wise linear function, stored in $X,Y_{\text{start}}$, and $Y_{\text{end}}$ arrays, and a *prefix lookup table D*. In this section, we will describe the data structure and, in doing so, prove the following Theorem:

**Theorem 1.** *Let* S *be a sorted list of N k-mers, let* ε≥1 *be a positive integer, and let* 0<ℓ<1 *be a real number. There exists a data structure called the basic PLA-index with the following properties*.

- *It can be constructed in* Θ(N) *time*.
- *The total bits used is* $b\left(2k-lg\left(\frac{b^{2-\ell }}{N(1+4\varepsilon )}\right)+c\right)+o(b)$, *where b is a function of* S *and* ε, *and c is a value between 3 and 4*.
- *It supports the rank and search operations in* Θ(lgb+lgε) *time*.

### 3.1 Construction

We process the *k*-mers of S from smallest to largest, treating each *k*-mer as an integer between 0 and $4^{k}-1$. For each *k*-mer x∈S, we define its y-range to be [rank(S,x)−ε,rank(S,x)+ε]. We feed *x* and its y-range to O’Rourke’s algorithm, until the algorithm stops and outputs a line that fits the previously given ranges. We store the *k*-mer and the y-value of the first point of this line in X and $Y_{\text{start}}$, respectively. We store the y-value of the last fitted point in $Y_{\text{end}}$. We then restart O’Rourke’s algorithm, but we reuse the last *k*-mer of the previous fitted line to start the next iteration. As will be clear later, this will allow us to more compactly represent the $Y_{\text{end}}$ array. The construction algorithm runs in Θ(N) time. Memory use is linear with respect to the maximum number of *k*-mers in a fitted line and is inversely related to ε.

In order to store the $Y_{\text{start}}$ and $Y_{\text{end}}$ values as integers, rather than reals, we round the y-values returned by O’Rourke’s algorithm. In some rare cases, this results in a line that no longer fits the range of some point *x*. In these cases, we re-run O’Rourke’s algorithm but manually terminate it at *x*, using whatever line is a valid fit up to that point. We then restart from *x*. Our experimental results will show that, in practice, the amount of such forced breaks is negligible (Section 6).

We will let *b* denote the number of lines created by the construction algorithm, i.e. the number of segments in our PLA. This is also the number of elements in $X,Y_{\text{start}}$, or $Y_{\text{end}}$.

We also construct a prefix lookup table D, which is used to speed up binary search on X. We have a parameter 0<ℓ<1 that trades off the size of D and the average search range in X. We set ℓ automatically to be roughly 1/16, subject to the constraint that bℓ is a power of two (formally, $\ell =\frac{2^{\left\lfloor lg\;b\right\rfloor }}{16b}$). D contains bℓ entries, each of size ⌈lgb⌉ bits, and the *i*^th^ element of D is the smallest position *j* such that the first lg(bℓ) bits of X[j] are at least *i*. This results in an average search range in X of 1/ℓ entries, while the size of D is ℓb⌈lgb⌉ bits. D can be trivially constructed by a linear scan through the X array.

### 3.2 Queries

**Figure 2:**
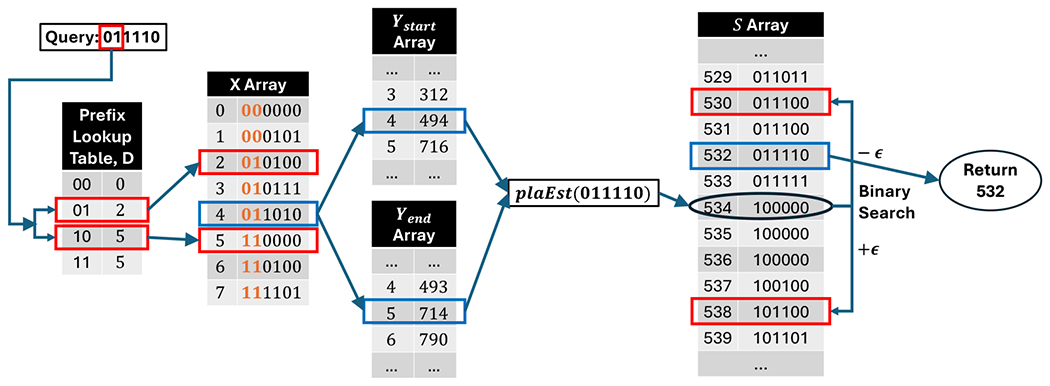
Example of a search query using the PLA-index. The query is $x=(011110{)}_{2}=(30{)}_{10}$ and we set ε=4 and bℓ=4. Each of the arrays is shown with two columns, where the left column shows the indices and the right column shows the array values. The indices of D are shown in binary, while the rest of the tables’ indices are shown in decimal. The values of the X and S arrays are shown in binary, while the rest of the tables’ values are shown in decimal. To perform the query, we start by extracting the leftmost y=lgbℓ=2 bits of the query. In our case, these bits have the value $(01{)}_{2}=(1{)}_{10}$. We then look up the value of D[1] and the value following that, D[2]. We then do a binary search in X between the values stored at D[1] and D[2], i.e. between locations 2 and 5 in X. We find that the largest index *i* such that X[i]≤x is $4\left(X[4]=(011010{)}_{2}=(26{)}_{10}\right)$. Thus, we know that if *x* is present, it must be in the $4^{th}$ segment. The ending x-value of this segment is $X[5]=(110000{)}_{2}=(48{)}_{2}$. The y-values that start and end this segment are found in $Y_{\text{start}}[4]$ and $Y_{\text{end}}[5]$. With these values in hand, we can calculate $\text{plaEst}(30)=Y_{\text{start}}[4]+\frac{Y_{\text{end}}[5]-Y_{\text{start}}[4]}{X[5]-X[4]}(x-X[4])=494+\frac{714-494}{48-26}(30-26)=534$. Finally, we do a binary search in S[⌊534−4⌋],…,S[[534+4⌉]. At position 532, we find that $S[532]=(011110{)}_{2}=(30{)}_{10}=x$. We return this position as search result.

Let *y* be the first lgbℓ bits of *x*, viewed as an integer. This gives us an index into D and we look up D[y] and D[y+1]. Then, we do a binary search in X between positions D[y] and D[y+1] to find the largest index *i* such that X[i]≤x. Then we can compute plaEst(x) according to Eq. 1. We then do a binary search in S[⌊plaEst(x)−ε⌋],…,S[⌈plaEst(x)+ε⌉] to find the smallest value *i* such that S[i]≥x. We return *i* if S[i]=x and return −1 otherwise The only difference between search and rank is that with search we can shortcut the binary search in S as soon as we hit a value *i* such that S[i]=x. Figure 2 illustrates how query works on an example.

The time to compute rank is the time to do a binary search on X plus the time to do a binary search on S. While the prefix lookup table speeds up the binary search on X in practice, in the worst case it can still be Θ(lgb). The binary search on S takes Θ(lgε) time. Thus the worst case total time for rank is Θ(lgb+lgε). This is no worse than index-less binary search (as long as bε≤N, which previous theoretical result suggest: Theorem 3.1 in [10]; Lemma 2 in [9]). The big advantage, however, is gained from cache effects, since in practice we observe that the PLA-index fits into the cache while S is stored in RAM. In this case, the number of RAM accesses now depends only on ε and not on N.

#### Compact storage

The arrays $X,Y_{\text{start}}$, $Y_{\text{end}}$, and D can be naïvely stored using 2kb,b⌈lgN⌉,b⌈lgN⌉, and ℓb⌈lgb⌉ bits, respectively. However, we exploit properties of the first three arrays to store them more compactly. The X array is an array of increasing integers, which can be represented compactly using the Elias-Fano technique [6, 7], as described in [23] and implemented in [20]. Elias-Fano encodes an array of m non-decreasing elements coming from a universe of size U in $m\left\lceil lg\;(U/m)+c_{\text{ef}}\right\rceil +o(m)$ bits, where $c_{\text{ef}}$ is a number between 1.5 and 2. It supports constant time access to arbitrary elements. In our case, the space for the x-values is $b\left\lceil lg\;\left(4^{k}/b\right)+c_{\text{ef}}\right\rceil +o(b)$ bits.

Elias-Fano lookups are nevertheless slower than the naïve encoding in practice, and we query X repeatedly as part of computing plaEst. We therefore consider an alternate encoding in practice. Consider the *i*^th^ element and let x=X[i]. If the values of X were distributed evenly among all the universe of 4^*k*^ *k*-mers, then *x* would be $i4^{k}/b$. Instead of storing *x*, we store the difference between *x* and this value, i.e. we store $x-i4^{k}/b$. To the extent that the *k*-mers of X are somewhat evenly distributed among the universe, the stored difference is small. This allows us to use a small number of bits to store each value. We use a constant-width encoding, where the number of bits is chosen so that it can fit the largest difference in X. We find in practice that this takes more space than Elias-Fano but less space than the naïve 2k bit encoding, while performing lookups as fast as with the naïve encoding.

To compact the $Y_{\text{start}}$ array, we first show that its values are non-decreasing. The following lemma gives the basis for this.

**Lemma 2.** *Given a line fitted by O’Rourke’s algorithm, let* $y_{\text{start}}$ *denote the* *y*-*value of the line at the first fitted point and let* $y_{\text{end}}$ *denote the* *y*-*value of the line at the last fitted point. Let* $X_{fit}=\left(x_{start},\ldots ,x_{end}\right)$ *be the sequence of* *k*-*mers that are covered by a run of O’Rourke’s algorithm during the PLA-index construction algorithm. Then there exists a line that fits* $X_{\text{fit}}$ *such that*


(2) $$y_{\text{start}}\leq \text{rank}\left(S,x_{\text{end}}\right)-\varepsilon \;\;y_{\text{end}}\geq \text{rank}\left(S,x_{\text{start}}\right)+\varepsilon$$

**Proof.** Observe that if $rank\left(S,x_{\text{start}}\right)+\varepsilon \leq rank\left(S,x_{\text{end}}\right)-\varepsilon$ then any fitted line will satisfy the lemma. Therefore, we assume that $rank\left(S,x_{\text{end}}\right)<rank\left(S,x_{\text{start}}\right)+2\varepsilon$. Consider the line with $y_{\text{start}}=rank\left(S,x_{\text{end}}\right)-\varepsilon$ and $y_{\text{end}}=rank\left(S,x_{\text{start}}\right)+\varepsilon$. Let *x* be an element of $\left(x_{\text{start}},\ldots ,x_{\text{end}}\right)$ and let *y* be the value of this line at *x*. By our assumption, the line has positive slope, and, therefore, $y_{\text{start}}\leq y\leq y_{\text{end}}$. Then,

$$\text{rank}(S,x)\leq \text{rank}\left(S,x_{\text{end}}\right)=y_{\text{start}}+\varepsilon <y+\varepsilon \;\text{rank}(S,x)\geq \text{rank}\left(S,x_{\text{start}}\right)=y_{\text{end}}-\varepsilon \geq y-\varepsilon$$

**Figure 3:**
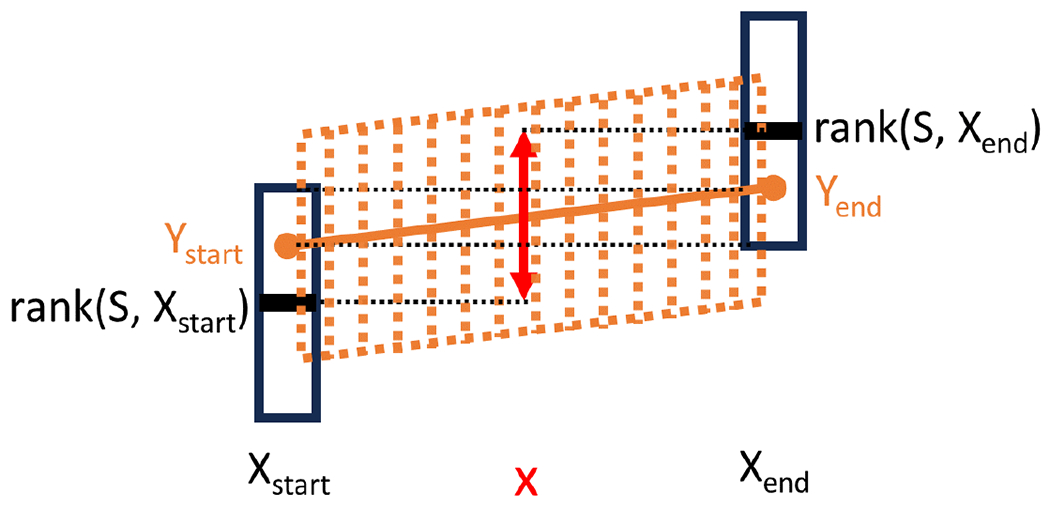
Illustration of the proof of Lemma 2. The values of $rank\left(S,x_{\text{start}}\right)$ and $rank\left(S,x_{\text{end}}\right)$ are shown in black, along with the ±ε vertical region around them. The proposed fitting line is shown in solid orange, and the dashed region denotes the ±ε region covered by the line. A sample middle point *x* is shown in red and its possible rank(S,x) values are denoted by the range of the red arrow. The proof is based on the fact that the red range is covered by the orange range.

Therefore, the line covers *x* and hence fits $X_{\text{fit}}$. Figure 3 illustrates the idea of the proof.

Lemma 2 can be used to guarantee that both $Y_{\text{start}}$ and $Y_{\text{end}}$ are non-decreasing, but our encoding only needs that $Y_{\text{start}}$ is non-decreasing. To guarantee that the line chosen by O’Rourke’s algorithm satisfies the $y_{\text{start}}$ constraint (Eq. 2), we can choose the line with the smallest $y_{\text{start}}$ value. We now prove that $Y_{\text{start}}$ is non-decreasing:

**Corollary 3.** *Let* 0≤i<b−1. *Then* $Y_{\mathrm{start}}[i]\leq Y_{\mathrm{start}}[i+1]$.

**Proof.** Let *x* be the last *k*-mer fitted by segment *i* (i.e. the segment starting at X[i]). Because *x* is the end of segment *i*, Lemma 2 gives that $Y_{\text{start}}[i]\leq rank(S,x)-\varepsilon$. In the way that we use O’Rourke’s algorithm during construction, we have that x=X[i+1]. However, all that is needed for the proof is that x≤X[i+1] and, since the rank function is increasing, rank(S,x)≤rank(S,X[i+1]). Therefore, $Y_{\text{start}}[i]\leq rank(S,X[i+1])-\varepsilon$. Simultaneously, since segment i+1 must fit X[i+1], we have that $Y_{\text{start}}[i+1]\geq rank(S,X[i+1])-\varepsilon$. Hence, we get $Y_{\text{start}}[i]\leq rank(S,X[i+1])-\varepsilon \leq Y_{\text{start}}[i+1]$.

Therefore, we can encode the $Y_{\text{start}}$ values using Elias-Fano. $Y_{\text{start}}$ contains b elements from a universe of size N, so the space used is $b\left\lceil lg\;(N/b)+c_{ef}\right\rceil +o(b)$ bits. Note also that the $Y_{\text{start}}$ values are only accessed once during a rank operation, to compute the slope of the segment. Therefore, the slower access time of Elias-Fano does not have a substantial effect on runtime.

To compact the $Y_{\text{end}}$ array, we encode each value relative to $Y_{\text{start}}$. Consider an arbitrary position *i* in $Y_{\text{end}}$ and let x=X[i] be the *k*-mer at *i*. Recall that the segment starting at X[i−1] ends at *x* with a y-value of $Y_{\text{end}}[i]$, while the segment starting at *x* starts with a y-value of $Y_{\text{start}}[i]$. Both segments are subject to the max error constraint at *x* so therefore $\left|Y_{\text{end}}[i]-rank(S,x)\right|\leq \varepsilon$ and $\left|Y_{\text{start}}[i]-rank(S,x)\right|\leq \varepsilon$. Putting it together, $-2\varepsilon \leq Y_{\text{end}}[i]-Y_{\text{start}}[i]\leq 2\varepsilon$. Therefore, we can encode $Y_{\text{end}}[i]$ by encoding the difference with $Y_{\text{start}}[i]$, using only ⌈lg(1+4ε)⌉ bits per entry. Note that this is why we run O’Rourke’s algorithm starting from the previous fitted *k*-mer. This guarantees that we can efficiently encode the difference between $Y_{\text{start}}$ and $Y_{\text{end}}$ using a fixed number of bits.

We can now derive the total bits used by the basic PLA-index, as stated in Theorem 1. We assume that X is stored using Elias-Fano and, for convenience, we ignore ceilings. The total space used by $X,Y_{\text{start}},Y_{\text{end}}$, and D is:

$$\left(b\left(\text{lg}\frac{4^{k}}{b}+c_{\text{ef}}\right)+o(b)\right)+\left(b\left(\text{lg}\frac{N}{b}+c_{\text{ef}}\right)+o(b)\right)+(b\text{lg}(1+4\varepsilon ))+\ell b\;\text{lg}\;b\;=b\left(\text{lg}\frac{4^{k}}{b}+\text{lg}\frac{N}{b}+\text{lg}(1+4\varepsilon )+\ell \;\text{lg}\;b+2c_{\text{ef}}\right)+o(b)\;=b\left(2k-\text{lg}\left(\frac{b^{2-\ell }}{N(1+4\varepsilon )}\right)+2c_{\text{ef}}\right)+o(b).$$

#### Choosing ε

If the user has in mind how large of an error their runtime can tolerate, they can set ε directly. Alternatively, they can set a target memory for the index. In this case, we can run the construction algorithm and compute the ratio r=N/b, using an arbitrary ε, e.g. ε=256. If we make the simplifying assumption that N/b does not vary greatly as a function of ε, then we can replace N with rb in the space equation given by Theorem 1, and then solve it (ignoring the lower order o(b) term) to obtain the value of ε that would give the target memory. This approach is not very precise but can get the index in the ballpark of the target memory. More sophisticated techniques have been presented in [10], but we have not implemented them in our prototype.

## 4 PLA-index with repeat stretching

In this section, we describe an alternative version of the PLA-index which is preferable when the common query is search rather than rank and S contains a lot of repeats. The idea is that if we are allowed to report any position containing *x*, rather than necessarily the first one, then we can allow plaEst(x) to be ε higher than the last (rather than the first) occurrence of *x*. In this way, we can give more leeway to O’Rourke’s algorithm, allowing it to use fewer segments.

### 4.1 Construction and storage

Let occ(x) define the number of times a *k*-mer is repeated in S. We modify the basic PLA-index construction algorithm by modifying the y-ranges fed to O’Rourke’s algorithm, making them [rank(S,x)−ε,rank(S,x)+ε+occ(x)−1]. In other words, we expand the y-range so that any y-value in the range is at most ε away from some position of *x*, but not necessarily the first one. The rest of the construction and storage is identical to the basic PLA-index, except for the two aspects we describe below.

When using repeat stretching, we can no longer guarantee that $Y_{\text{end}}[i]$ and $Y_{\text{start}}[i]$ values lie within 2ε of each other, because occ(*x*) can be as high as N−n+1. Nevertheless, we observe that $\left|Y_{\text{end}}[i]-Y_{\text{start}}[i]\right|$ still tends to be small. We therefore can encode $Y_{\text{end}}$ using a technique for variable-width encoding of integers that allows random access, known as Directly Addressable Codes [4] and implemented in [11]. Since the encoding only works for non-negative values, we transform the values prior to encoding to be $2\left|Y_{\text{end}}[i]-Y_{\text{start}}[i]\right|+t$, where t=1 if $Y_{\text{end}}[i]-Y_{\text{start}}[i]$ is positive and t=0 otherwise.

The reason that the basic PLA-index construction algorithm reused the previously fitted *k*-mer for O’Rourke’s algorithm was to guarantee that $Y_{\text{end}}[i]-Y_{\text{start}}[i]$ is bounded. Since this is no longer possible, we now do not reuse the *k*-mer, thereby decreasing the number of segments. Further, the proof of Corollary 3 also works without the *k*-mer reuse, and so we can encode $Y_{\text{start}}$ as before.

### 4.2 Queries

The algorithm for the search query remains the same as for the basic PLA-index. To understand the runtime of rank, we distinguish between two ways that S may be represented, which affects the cache locality of accessing consecutive elements. In the *indirect* case, S is represented indirectly via pointers. For example, let S be the sorted *k*-spectrum of a genome. Then the suffix array of the genome is an indirect representation of S (ignoring suffixes shorter than *k*). That is, accessing the suffix array at location *i* gives you a location in the genome that is the start of the *k*-mer S[i]. In such a situation, accessing consecutive *k*-mers of S is not cache-local, because consecutive *k*-mers in S are not necessarily nearby in the genome. In the *direct* case, S is represented directly, e.g. an array of *k*-mers. In such a case, it is not necessary to look into the genome and accessing consecutive values of S becomes fast due to cache locality.

Now, to compute rank(*x*), we first let *p* = search(*x*). Then, to get rank(S,x), we continuously decrement p as long as S[p]=x. If S is stored directly, then rank is fast because of cache locality. If S is stored indirectly, then each access to a *k*-mer of S incurs a cache miss, making rank slow. This can be sped up by storing an additional bitvector, B, which marks the positions in S which have a *k*-mer that is different from the preceding *k*-mer. However, this adds N bits of space, which can easily dwarf the space of the PLA-index. We therefore do not recommend using repeat stretching for the case that rank needs to be supported and S is stored indirectly.

## 5 PLA-index-exact

The basic (and repeat-stretched) PLA-index can be viewed as using a tiny bit more space to significantly speed up binary search. However, it still does not perform as fast as a direct access table of *k*-mer ranks. We therefore propose a variant of the PLA-index that is as fast as the direct access table of *k*-mer ranks but takes substantially less space. A similar idea was used in the context of a PLA-based dictionary [3].

First, we construct an MPHF with the set of keys being the distinct *k*-mers in S. We then build the basic PLA-index. Finally, we construct an error array E of size n. For each distinct *x* in S, we set E[MPHF(x)]=plaEst(x)−rank(S,x). To perform rank(*x*), we let p=plaEst(x)−E[MPHF(x)], check if S[p]=x, and if yes, then return p, otherwise return −1. The binary search through S done by the basic PLA-index is now avoided and replaced with the cost of one MPHF calculation and one access to E.

Since each entry in E is guaranteed to be between −ε and +ε, the additional space required over the basic PLA-index is nlg(2ε+1) bits for the E array and the space to store the MPHF (usually 2−4 bits per *k*-mer). The additional space is decreased for lower ε but the space of the basic PLA-index is increased (Theorem 1). We explore the trade-off in Section 6.4.

## 6 Experimental results

In this section, we evaluate the performance of the PLA-index and demonstrate how it can be applied in a variety of bioinformatic applications. PLA-index is freely available and opensource at https://github.com/medvedevgroup/pla-index, along with reproducibility information for the experiment in this section.

### 6.1 Experimental setup

We used a machine with an Intel(R) Xeon(R) CPU E5-2683 v4 @ 2.10GHz processor with 64 cores and 512 GB of memory to run our experiments. Unless otherwise stated, all reported running times are wall clock times and medians of five runs. The results of all search and rank PLA-index operations were confirmed for correctness. We note that our experiments with the PLA-index do not change the output of any downstream applications, thus we do not report accuracy in any of the experiments. Unless otherwise stated, we used k=21 for our indices, in line with previous work [15]. We used libdivsufsort [18] to construct suffix arrays and we use PTHash [22] for MPHF construction.

### 6.2 PLA-index speeds up suffix array queries

We demonstrate how the repeat-stretched PLA-index can be used to speed up the search query in the case that S is represented indirectly via a suffix array. We compare against an index-less binary search, Sapling [15], and two versions of the PGM-index [9]. There are other *k*-mer indexing tools (e.g. SSHash [21]), including ones using piece-wise linear approximation to the rank curve (eg. Lemonhash [8]); however, as these are not search indices, we do not compare against them. We use three genomes: the human genome (hg38, *N* = 3, 049, 315, 763, *n* = 2, 333, 046, 826), the Gorilla genome (RefSeq Accession GCF_029281585.1, *N* = 3, 595, 314, 193, *n* = 2, 346, 463, 274), and the *C.elegans* genome (RefSeq Accession GCF_000002985.6, *N* = 100, 286, 381, *n* = 93, 046, 063).

**Figure 4:**
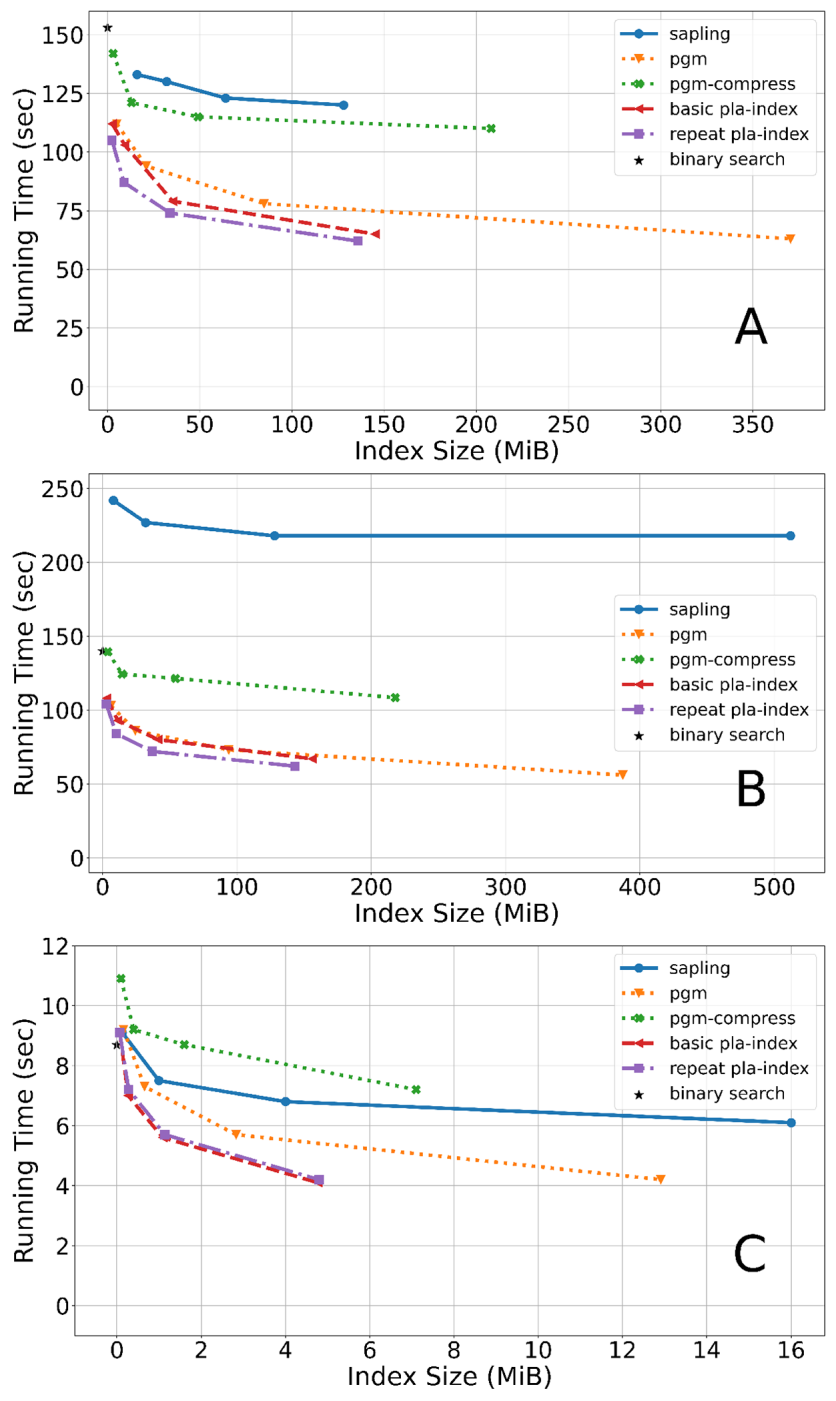
Suffix array search query time on the whole genomes of human (Panel A), Gorilla (Panel B), and *C.elegans* (Panel C). To measure query time for a genome, we randomly choose 50 million positions of the genome (for *C.elegans* we chose 5 million), and used the *k*-mers (k=21) starting at those positions as queries. We report the total wall-clock time needed to run all the queries. PGM-index only returns an interval of possible locations, so we supplemented it with the same binary search as we have in PLA-index. Binary search does not use an index, hence the running time is shown at index size 0. Each curve was generated from four runs. For non-Sapling tools, the runs corresponded to values of ε∈{15,63,255,1023}. For Sapling, the runs corresponded to setting the number of buckets to $2^{20},2^{21},2^{22}$ and $2^{23}$ for human, $2^{19},2^{21},2^{23}$ and $2^{25}$ for Gorilla, and $2^{13},2^{16},2^{18}$ and $2^{20}$ for *C.elegans*. Raw numbers are shown in the Appendix in Tables 5, 6, and 7.

For the human genome (Figure 4A), first, we observe that the repeat-stretched PLA-index outperforms the basic PLA-index. Second, the (repeat-stretched) PLA-index nearly halves the time of a regular binary search when used with ε=63, for only 34 MiB of space. Third, when compared to Sapling, for similar index size, PLA-index is at least 75% faster (a precise comparison is difficult because the index sizes do not match exactly). This underscores the importance of an error guarantee, optimal segment selection, and compact representation, as these are the main improvements of PLA-index over Sapling. Fourth, the PLA-index uses less than half the space of the PGM index, when keeping the query time fixed.

For the Gorilla (Figure 4B) and *C.elegans* (Figure 4C) genomes, the trends of the results are generally the same. A major difference is that for *C.elegans*, the basic and repeat-stretched PLA-index have near identical performance. This can be explained by the fact the average repeat length in *C.elegans* is N/n=1.08, while for the human it is N/n=1.31. As expected, repeat stretching does not help when there are not many repeats.

Overall, we find it remarkable how small the PLA-index is. With only 2 MiB of memory, we can estimate the position of a *k*-mer in a suffix array of size ≈3 billion to within 1023 positions. With 34 MiB of memory, we can estimate it to within 63 positions.

We also measured construction time and memory on hg38 (Table 1). The PLA-index was roughly the same as the PGM-index: it used a little bit less memory but was a little bit faster. Sapling was substantially slower and more memory intensive. Overall, we did not optimize construction time or memory, so we believe there is a lot of room for improvement.

**Figure 5:**
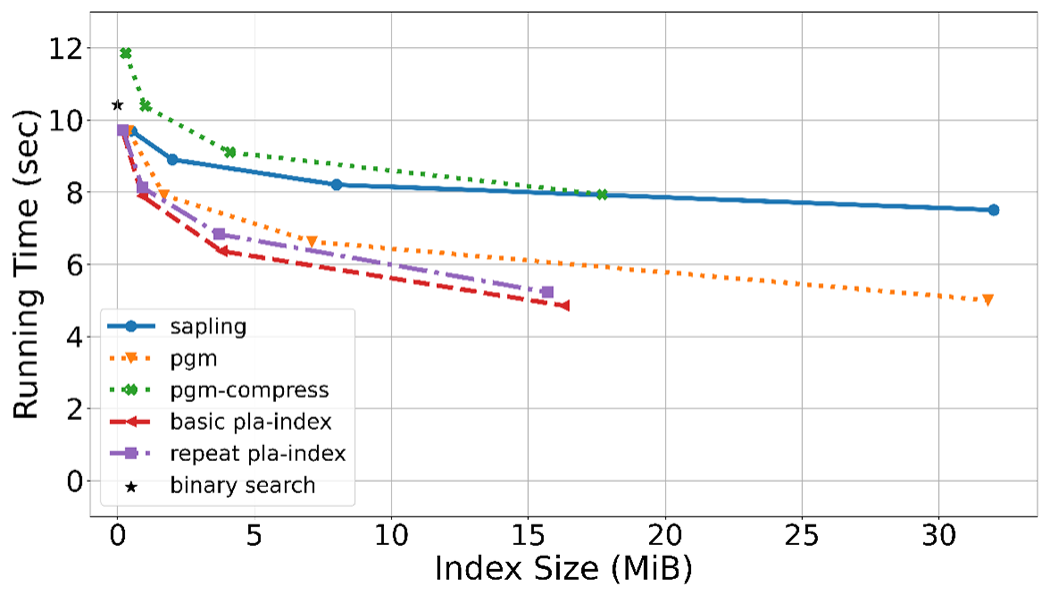
Suffix array search time on human chromosome 1 using k=31. We used the same experimental setup as Figure 4, but, because chr1 is relatively small, we used only 5 million queries and set ℓ=1/8. For Sapling, the runs correspond to setting the number of buckets to $2^{15},2^{17},2^{19}$ and $2^{21}$.

We also evaluated the performance of PLA-index for a different *k*-mer size (k=31) and found the resulting trends to be the same as for the chromosome 1 of the whole genome (Figure 5).

### 6.3 PLA-index reduces memory use of read aligner

Strobealign is a recent aligner for short reads [26]. To represent the reference, it uses a seed table where each row corresponds to a seed and contains the 64-bit hash of the seed sequence and its associated data. The rows are sorted in increasing order of hash values. Thus, the seed table is a direct representation of a sorted list of values, with the minor difference that each element is not a *k*-mer but a 64-bit hash value. To align the reads, strobealign repeatedly searches the table to find the location of a read’s seeds in the reference. In order to avoid cache-unfriendly binary search, strobealign also stores a large pointer vector (e.g. for the human reference, it has 2^28^ elements), where the element at position *h* is the index of the first row in the seed table whose hash value starts with *h*.

We modified strobealign by replacing the pointer vector with the repeat-stretched PLA-index. Table 2 shows that the PLA-index takes two or three orders of magnitude less space than the pointer vector. For example, while the pointer vector takes 2 GiB for the human, PLA-index takes 1.5 MiB for ε=63. The overall memory usage of strobealign is still dominated by other components of their index; though these can be substantially optimized [27], it is outside the scope of our project. We did observe a 1% slow down on Drosophila and about a 5% slow down on human. We believe that this is simply due to the fact that strobealign code has been highly optimized for speed [27], while our implementation is only a prototype. We note that increasing ε does not increase the runtime, indicating that the overhead of implementing rank on a repeat-stretched PLA-index when S is stored directly is negligible.

### 6.4 PLA-index-exact reduces memory of a direct access rank table

We compare PLA-index-exact to the fastest alternative option, which stores each *k*-mer’s exact rank, rather than its error. Table 3 shows that PLA-index-exact offers a drastic improvement, reducing the total space by 76% without increasing the run time (using ε=63 on hg38). Further space improvements are possible by making ε=15, but the run time starts to increase beyond that of the direct access rank table. PLA-index-exact also more than halves the search time of the repeat-stretched PLA-index (Figure 4A), though at a considerable space cost. Table 3 also shows that decreasing ε does not speed up the search time; the run time seems to be influenced by low-level system effects, and we do not pursue the question further in this paper.

### 6.5 Number of segments

The intention behind repeat stretching is that it should reduce the number of segments. Table 4 confirms this effect in practice. Using ε=63 for illustrative purposes, the percentage decrease in the number of segments is 11%, 16%, and 1%, respectively for Human, Gorilla, and *C.elegans*. Unsurprisingly, this correlates with how repetitive the genomes are. In particular, the N/n values for these three genomes are 1.31,1.53, and 1.08, respectively.

Table 4 also confirms that the the number of forced segments created by our construction algorithm is negligible, i.e. about 1 in 10,000 in the worst case. Thus the idea of improving compression by rounding the $Y_{\text{start}}$ and $Y_{\text{end}}$ values does not result in any noticeable increase of the number of segments.

## 7 Conclusion

In this paper, we presented several variations of the PLA-index, a search index for a sorted list of *k*-mers. The PLA-index exploits the linear-like structure of the rank curve in order to speed up rank and search queries. It uses an order of magnitude less space than Sapling and uses less than half the space of the PGM-index, for roughly the same query time. We demonstrated how the PLA-index can be applied in various settings to achieve dramatic time and/or memory improvements. For example, the PLA-index sped up the binary search for a *k*-mer in a suffix array by two-fold, reduced the memory of a short-read aligner by 2 GiB on human dataset, and reduced the memory of a direct access table of *k*-mer ranks by 76%.

One can imagine different ways in which the rank curve linearity can be exploited to index sorted lists. The PLA-index is just one possibility, guided by our own design choices. However, several reasonable alternatives might be pursued. For example, one can abandon the maximum error guarantee of the piece-wise linear function and instead take a heuristic or probabilistic approach to reducing the error (e.g. reduce the average error). One might also not be limited by a linear function but instead fit quadratic function, or, even better, learn from the data a set of functions that can be fit to each piece. While some alternatives were explored in previous works (e.g. [15]), we believe there remains a lot of unexplored potential.

More broadly, the linearity of the rank curve of genomic spectra could be exploited by other data structures and algorithms. For example, [3] proposed a PLA-based dictionary, but it has not been applied in the *k*-mer setting. Other possibilities include minimum perfect hash functions [8], rank and select data structures, and *k*-mer counting. We believe that PLA-based approaches have the potential to outperform many state-of-the-art approaches when dealing with genomic *k*-mer data.

## Figures and Tables

**Table 1:** Construction time and memory for hg38. Index size is shown in MiB, construction time is shown in minutes, and construction memory is shown in GiB. Peak memory was measured.

Basic PLA-index			Repeat PLA-index			PGM-index			Sapling		
Index	Construction		Index	Construction		Index	Construction		Index	Construction	
Size	Time	Mem	Size	Time	Mem	Size	Time	Mem	Size	Time	Mem
2.5	21	26	2.4	23	26	5.0	19	29	16	35	100
9.3	21	26	8.9	22	26	21.0	19	29	32	33	100
35.3	21	26	33.8	21	26	84.9	19	29	64	29	100
145.1	21	26	135.8	20	26	370.4	20	29	128	32	101

**Table 2:** Breakdown of memory usage and runtime by Strobealign, with and without our PLA-index. The PV column indicates the space used by the Pointer Vector, which our modified version replaces with PLA-index. The “Other” column refers to the sum of all other components of the aligner that remain unaffected by our modifications. To measure runtime, we used 16 threads and measure wall clock time of a single run. The Drosophila experiment uses a read set with 10.5 million paired-end reads and BDGP6.22 [24] as the reference. The human experiment uses a read set with 10 million paired-end reads with T2T-CHM13v2.0 [25] as the reference. All reads were simulated using the same setup as [26].

			Index memory (MiB)			Alignment time (s)	
Dataset	Max Error	N. segments	PLA-index	PV	Other	Modified	Original
			
Drosophila	255	495	.01	64	615	421	417
	63	6,368	.07			427	
	15	62,092	.60			425		

Human	255	28,374	0.3	2,048	12,710	630	590
	63	136,322	1.5			622	
	15	1,055,142	10.5			625	

**Table 3:** Performance of PLA-index-exact on the suffix array of hg38 on 50 million queries. We compare against storing the exact ranks in a direct access table. The run-times reported are the medians of five independent runs. The queries are the same as in Figure 4A.

		PLA-index-exact			Rank table	
Max error	MPHF (MiB)	PLA-index (MiB)	Table (MiB)	search time (s)	Table (MiB)	search time (s)
4,095	728	0.6	3,616	27	8,900	32
1,023		2.5	3,059	28		
255		9.3	2,503	28		
63		35.3	1,947	31		
15		145.1	1,391	33		

**Table 4:** The number of segments in the basic and repeat-stretched PLA-indices, for the three genomes in Figure 4. The number of forced segments is the number of segments that need to be added because we round $Y_{\text{start}}$ or $Y_{\text{end}}$.

Genome	Max error	Number of segments			
		Basic		Repeat-stretched	
		Total	Forced	Total	Forced
Human	1,023	302,990	1	269,717	16
	255	1,198,397	1	1,066,934	88
	63	4,831,533	5	4,290,786	465
	15	21,298,004	73	18,497,919	2,177

Gorilla	1,023	377,156	0	319,179	40
	255	1,461,437	1	1,226,663	179
	63	5,673,700	3	4,704,707	715
	15	22,932,639	102	19,156,813	2,719

*C. elegans*	1,023	8,039	0	8,022	0
	255	33,974	0	33,771	0
	63	148,427	0	144,405	0
	15	694,719	0	654,870	5

## Footnotes

1 It was brought to our attention after our algorithm was finalized that some of these ideas are also implemented in the code base of [8], though they are not described in the respective paper.

2 Note that rank returns −1 for elements not in S. Support for rank on elements not in S could be added to our indices, though we have not implemented it and therefore do not describe the details.

3 The crux of O’Rourke’s algorithm is how to maintain this set efficiently, but since we use the algorithm in a black-box manner, we omit this description.

## A Appendix

The Appendix contains Tables 5, 6, and 7, which provide the raw numbers used to generate Figure 4.

**Table 5:** Index sizes, in MiB, of PLA-index and PGM. This table provides the raw numbers already shown in Figure 4.

Genome	Max error	PLA-index		PGM	PGM-compress
		Basic	Repeat-stretched		
Human	1,023	2.5	2.4	5.0	3.3
	255	9.3	8.9	21.0	12.5
	63	35.3	33.8	84.9	49.0
	15	145.1	135.8	370.4	208.0

Gorilla	1,023	3.1	2.8	6.3	3.9
	255	11.3	10.2	24.4	14.5
	63	41.2	36.9	94.0	54.3
	15	156.0	143.2	387.2	217.9

*C.elegans*	1,023	0.07	0.07	0.16	0.11
	255	0.27	0.28	0.66	0.41
	63	1.09	1.14	2.83	1.65
	15	4.77	4.80	12.91	7.14

**Table 6:** Query times, in seconds, of PLA-index and PGM. This table provides the raw numbers already shown in Figure 4.

Genome	Max error	PLA-index		PGM	PGM-compress	Binary Search
		Basic	Repeat-stretched			
Human	1,023	112	105	112	142	153
	255	103	87	94	121	
	63	79	74	78	115	
	15	65	62	63	110	

Gorilla	1,023	108	104	103	139	140
	255	93	84	86	124	
	63	80	72	73	121	
	15	67	62	56	108	

*C.elegans*	1,023	9.1	9.1	9.2	10.9	8.7
	255	7.0	7.2	7.3	9.2	
	63	5.6	5.7	5.7	8.7	
	15	4.1	4.2	4.2	7.2	

**Table 7:** Sapling results. This table provides the raw size and time numbers already shown in Figure 4, along with the number of segments.

Genome	N. of segments	Index size (MiB)	Query time (s)
Human	1,048,576	16	133
	2,097,152	32	130
	4,194,304	64	123
	8,388,608	128	120

Gorilla	524,288	8	242
	2,097,152	32	227
	8,388,608	128	218
	33,554,432	512	218

*C.elegans*	8,192	0.13	9.1
	65,536	1.0	7.5
	262,144	4.0	6.8
	1,048,576	16.0	6.1
